# Retraction Note: The randomized clinical trial results of the anxiety treatment in patients with somatoform dysfunction and neurotic disorders

**DOI:** 10.1038/s41598-022-19938-3

**Published:** 2022-09-14

**Authors:** Vladimir Anatolevich Parfenov, Pavel Rudolfovich Kamchatnov, Dina Rustemovna Khasanova, Enver Ibragimovich Bogdanov, Tatiana Markovna Lokshtanova, Aleksandr Vitalevich Amelin, Natalya Nikolaevna Maslova, Nataliia Vyacheslavovna Pizova, Galina Nikolaevna Belskaya, Evgeny Robertovich Barantsevich, Gulsum Abdurahmanovna Duchshanova, Saltanat Ualihanovna Kamenova, Oleg Vladimirovich Kolokolov, Alexey Borisovich Glazunov

**Affiliations:** 1 I.M. Sechenov First Moscow State Medical University (Sechenov University), Moscow, Russia; 2 Pirogov Russian National Research Medical University, Moscow, Russia; 3 Kazan State Medical University, Kazan, Russia; 4 Pirogov Samara City Clinical Hospital №1, Samara, Russia; 5 Pavlov Medical University, Saint Petersburg, Russia; 6 Smolensk State Medical University, Smolensk, Russia; 7 Clinical Hospital №8, Yaroslavl, Russia; 8 Research Center of Neurology, Moscow, Russia; 9 Khoja Akhmet Yassawi International Kazakh-Turkish University, Turkestan, Kazakhstan; 10 Department of Medicine, Al-Farabi Kazakh National University, Almaty, Kazakhstan; 11 Razumovsky State Medical University, Saratov, Russia; 12 Pirogov Medical University, Moscow, Russia

Retraction of: *Scientific Reports* https://doi.org/10.1038/s41598-021-03727-5, published online 20 December 2021

The Editors have retracted this Article.

After publication of this Article concerns were raised regarding the design of the study and the robustness of its central conclusions. Post-publication peer review has confirmed that:

- there is insufficient justification for the grouping of the patients who belong to clinically heterogeneous cohorts with multiple different psychiatric disease presentations;
- the study lacks objective outcome measures; and
- there are concerns about the validity of the therapeutic intervention tested—specifically that Tenoten contains antibodies diluted beyond the point at which any active molecules are expected to be present and there is no molecular analysis to support the presence of molecules at these dilutions

The Editors therefore no longer have confidence in the conclusions presented.

Vladimir Anatolevich Parfenov, Dina Rustemovna Khasanova, Pavel Rudolfovich Kamchatnov and Alexey Borisovich Glazunov disagree with this retraction. Enver Ibragimovich Bogdanov, Tatiana Markovna Lokshtanova, Aleksandr Vitalevich Amelin, Natalya Nikolaevna Maslova, Nataliia Vyacheslavovna Pizova, Galina Nikolaevna Belskaya, Evgeny Robertovich Barantsevich, Gulsum Abdurahmanovna Duchshanova, Saltanat Ualihanovna Kamenova and Oleg Vladimirovich Kolokolov did not respond to correspondence from the Editors about this retraction.

